# Telomere Checkpoint in Development and Aging

**DOI:** 10.3390/ijms242115979

**Published:** 2023-11-05

**Authors:** Alla Kalmykova

**Affiliations:** Koltzov Institute of Developmental Biology, Russian Academy of Sciences, 119334 Moscow, Russia; allakalm@idbras.ru

**Keywords:** telomere, telomeric RNA, chromatin, germline, telomere dysfunction, lamins, aging, *Drosophila*

## Abstract

The maintenance of genome integrity through generations is largely determined by the stability of telomeres. Increasing evidence suggests that telomere dysfunction may trigger changes in cell fate, independently of telomere length. Telomeric multiple tandem repeats are potentially highly recombinogenic. Heterochromatin formation, transcriptional repression, the suppression of homologous recombination and chromosome end protection are all required for telomere stability. Genetic and epigenetic defects affecting telomere homeostasis may cause length-independent internal telomeric DNA damage. Growing evidence, including that based on *Drosophila* research, points to a telomere checkpoint mechanism that coordinates cell fate with telomere state. According to this scenario, telomeres, irrespective of their length, serve as a primary sensor of genome instability that is capable of triggering cell death or developmental arrest. Telomeric factors released from shortened or dysfunctional telomeres are thought to mediate these processes. Here, we discuss a novel signaling role for telomeric RNAs in cell fate and early development. Telomere checkpoint ensures genome stability in multicellular organisms but aggravates the aging process, promoting the accumulation of damaged and senescent cells.

## 1. Introduction

Linear chromosome ends are protected by nucleoprotein structures called telomeres, which maintain genome integrity through generations. Telomere research as a specific area of the life sciences originates from the theoretical works of Alexey Olovnikov written in 1971–1973, in which he described the phenomenon of the under-replication of chromosome ends, which he called marginotomy [1,2]. This phenomenon is associated with the inability of DNA polymerase to replicate the terminal DNA fragment, resulting in chromosome shortening with each cell cycle. According to experimental estimates, chromosome ends are shortened by 50 base pairs per human cell division and by 2–5 kilobases per generation [3]. Alexey Olovnikov predicted the existence of a specialized enzyme that compensates for the loss of terminal DNA and elongates the chromosome end in the endlessly dividing germline, stem, and tumor cells. The gradual shortening of terminal DNA during replication explains the Hayflick limit, which postulates a limited number of somatic cell divisions [4]. The telomere theory of aging proposed by Alexey Olovnikov explained how telomere shortening could be responsible for cell cycle arrest leading to replicative senescence of somatic cells. The limitation of cell doubling potential was considered by Alexey Olovnikov as a tumor suppression mechanism [2]. Moreover, in 1973, he formulated the idea that the “artificial lengthening of the telogenes could be a means of delaying ageing in proliferating cell clones” [2].

These visionary predictions were confirmed decades later by the discovery of telomerase, an enzyme that lengthens telomeres [5], and by studies demonstrating the effect of telomerase activation on the number of cell divisions [6] and the entire organism lifespan [7]. Studies of the organization and functioning of the exceedingly complicated telomere complex have revealed new aspects of telomere biogenesis and their link with aging. This area of research continues to be at the cutting edge of the life sciences 50 years after Olovnikov’s discovery. In this review, we will discuss the ways in which telomeres communicate with cell systems, since the consequences of this dialogue are extremely important for cell fate and development. The telomere checkpoint concept discussed here implies a pivotal role for telomere signaling in conditions threatening genome integrity.

## 2. A Telomere Is a Dynamic DNA-RNA-Protein Complex

**Figure 1:**
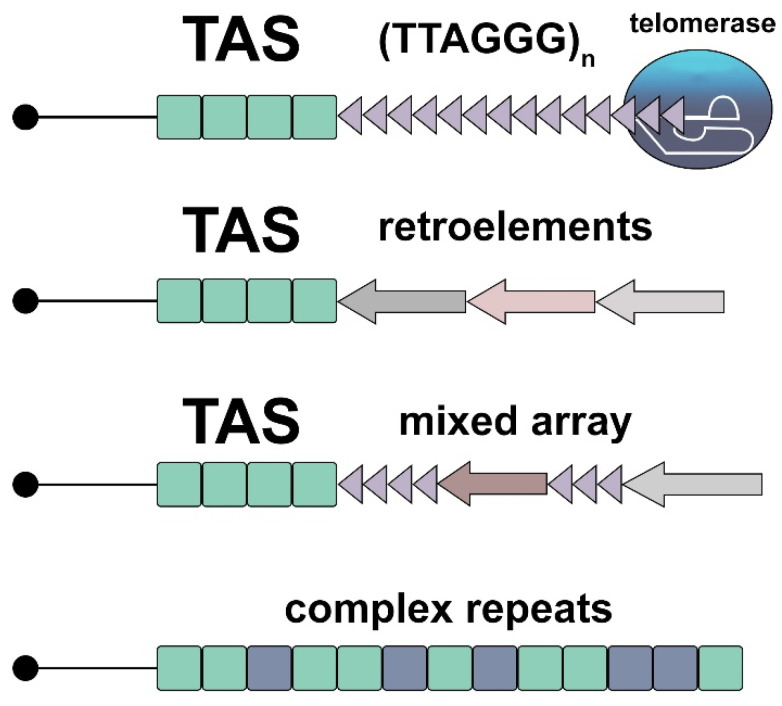
Different types of telomeric repeats. TAS, telomere-associated sequences. Arrowheads designate telomerase-generated repeats, different colored arrows show telomeric retrotransposons, different colored squares represent satellite-like repeats.

To understand how telomeres function in different cells, let us briefly recall how they are organized. Recent reviews provide detailed descriptions of the structure of telomeres [8,9,10]. The telomere complex includes telomeric DNA, a telomeric protective protein complex, and telomeric RNA, which forms an additional scaffold for binding telomeric proteins. There are several types of telomeric repeats and mechanisms for their multiplication. In most species, the mechanism of telomere lengthening is the reverse transcription reaction. The attachment of retroelements to the ends of linear DNA is thought to have evolved as the primary way of protecting linear chromosomes [11]. Telomerase is thought to be derived from a retroelement reverse transcriptase and later evolved to become a highly specialized enzyme for maintaining telomeres [12]. Using a template RNA (telomerase RNA component, TERC), telomerase reverse transcriptase (TERT) lengthens the ends of chromosomes by adding many short repeats, e.g., TTAGGG in mammals. Many Diptera insects, which are among the most prosperous and numerous species, have lost telomerase during evolution, returning to the most ancient way of maintaining telomeres: retrotransposon attachment to the chromosome ends [13,14,15]. Interestingly, in telomerase-encoding species, attachment of retrotransposons to telomeres is also observed, resulting in the formation of mixed-type telomeric repeats [16,17]. In mammals, the LINE1 retrotransposon is also able to attach to unprotected telomeres [18,19]. Finally, satellite-like complex repeats were found in mosquito telomeres, which are most likely maintained through recombination [20,21] (Figure 1). The study of this mechanism of telomere maintenance in natural populations can provide a new impetus to the search for ways of combating the most aggressive forms of cancer, whose telomeres are maintained using recombination or the alternative lengthening of telomeres (ALT) [22,23]. 

A special protein complex prevents the ends of chromosomes from being recognized as DNA double-strand breaks by the DNA damage response (DDR) and prevents recombination and chromosome fusion. The telomere protection complex in humans is termed shelterin and consists of six components: TRF1 (Telomeric repeat-binding factor 1), TRF2, RAP1, TIN2, POT1, and TPP1 [8,24]. A 3′ overhang generated at the chromosome ends as a result of incomplete end replication invades into a double-strand region forming a t-loop (telomeric loop). DNA binding proteins of shelterin interact with double- and single-strand telomeric DNA, protecting it from degradation and fusions. A functional analog of shelterin, the terminin protein complex, performs similar functions in *Drosophila* telomeres that are maintained by the retrotranspositions of telomeric retrotransposons [25]. In addition, functional telomeres are organized in heterochromatic domains with typical epigenetic marks [26]. It is believed that both telomere compaction and shelterin protein binding are required for the inhibition of ATM (ataxia telangiectasia mutated) and ATR (ataxia telangiectasia and Rad3-related) checkpoint kinases and the prevention of activation of DNA repair pathways at telomeres [24]. Shelterin proteins repress both nonhomologous end joining and homologous recombination at the telomeres to prevent telomere fusions and instability. Surprisingly, DNA repair factors interact with shelterin proteins to establish telomere end protection and telomere transcriptional silencing [27]. DNA repair factors are also required for the assembly of the telomere capping protein complex in *Drosophila*: ATM and ATR kinases and the Mre11-Rad50-Nbs1 (MRN) complex facilitate the loading of telomere protection and silencing proteins at *Drosophila* telomeres [28,29,30]. Most likely, DNA repair proteins transiently associate with telomere ends or specific telomeric factors to distinguish telomeres from dsDNA breaks and facilitate the binding of the telomere protection complex. The epigenetic control of telomeric chromatin is a complex process, which has not been fully understood to date and attracts particular attention due to its impairment in aging.

Despite the heterochromatic features of telomeres, they are transcriptionally active. Telomeric Repeat-containing RNA (TERRA) is a G-rich long non-coding RNA driven by the subtelomeric promoters [31]. TERRA biogenesis regulated by numerous nuclear factors resulted in the sequestration of the poly(A) minus TERRA fraction at the telomeres. Being able to interact with shelterin components, TERRA serves as a scaffold at the telomeres. TERRA is the most dynamic component of telomeres, the levels of which vary during the cell cycle and with changes in the state of telomeric chromatin. The functions of TERRA are being actively studied and it is already clear that telomeric RNAs have a huge regulatory potential. TERRA is involved in the formation of R-loops (RNA-DNA hybrids) and G-quadruplexes at the telomeres, both of which have regulatory significance [32]. It is believed that high levels of TERRA in cancer cells of the ALT type ensure telomere lengthening based on recombination [33]. 

A tight connection between the telomere and a network of cellular processes is suggested by the identification of numerous protein and RNP (ribonucleoprotein) partners of telomerase, components of shelterin complex and TERRA [34,35,36,37]. In-depth studies of the organization of telomeres show that this structure is both highly dynamic and multi-component, and many levels of regulation ensure the stable state of the telomere [8]. First of all, it must be kept in mind that the telomere consists of multiple tandem repeats, the same on all chromosomes, which carries an intrinsic recombinogenic danger, fraught with genetic instability. 

Telomere attrition is a significant contributor to aging [38,39]. The complexity and dynamism of telomere organization revealed in recent studies suggest that telomere length and telomerase activity are not the only parameters that may be associated with telomere dysfunction during normal development and organismal aging. In fact, the relationship between telomere length and chronological age in humans and 98 species of non-human vertebrates, as determined by large sample sizes, is weak and varies based on tissue types and telomere measurement methodology [40,41]. In addition to critically short telomeres, long but defective telomeres contribute to cellular senescence and age-related diseases, which are particularly noticeable in post-mitotic tissues [42,43,44]. 

## 3. The Signaling Role of Telomeric RNA

The disruption of the integrity of the telomere complex can lead to genetic instability, aneuploidy, chromosomal abnormalities, and, ultimately, neoplastic transformation. In a multicellular organism, cells with telomere dysfunction are eliminated, which serves as an important anti-oncogenic mechanism in the development. Therefore, there must be mechanisms for the transmission of signals from dysfunctional telomeres to cellular processes. Such a mechanism is easy to imagine, given the numerous structural and functional relationships between telomere components and biochemical and cell signaling pathways. According to the present evidence, there are two main signaling pathways from the telomeres. The first is the release of telomeric proteins or RNA from the telomeres under certain conditions and their effect on cellular targets. The second is the accumulation of damage at the telomeres above the threshold level, which activates DNA repair systems and then the mechanisms of cell death. In this review, we will consider both mechanisms as part of a single telomere checkpoint pathway.

The mechanisms of telomere signaling concerned with the regulation of non-telomeric targets by telomeric proteins are described in comprehensive reviews [45,46,47]. The most illustrative is the budding yeast model. During the shortening of yeast telomeres, the average length of which is ~350 bp, telomeric proteins are released and interact with gene regulatory regions in the nucleus. For example, Rap1 relocalization from the telomeres correlates with the repression of core histone genes, which is typical for senescent cells [48]. Mammalian shelterin proteins and telomerase have also been implicated in the transcriptional regulation of non-telomeric genes [47]. Extratelomeric nuclear Rap1 regulates the expression of energy metabolism [49] and immunity-related genes [50]. Cytoplasmic Rap1 induces proinflammatory signaling, reducing lifespan in mice [51]. Thus, the signaling role of the telomeric Rap1 protein is evolutionarily conserved. It remains an open question whether the mechanism of release and relocalization of Rap1 in mammals is also determined by telomere shortening, as it is in yeast. If this is the case, then such a “titration” mechanism can explain the changes in the proliferative status of cells during replicative aging.

Telomerase reverse transcriptase, TERT, also regulates non-telomeric targets. For example, TERT occupies promoters and regulates the expression of genes involved in the Myc- and Wnt-signaling pathways via an interaction with the BRG1 chromatin remodeling protein [52]. By controlling gene expression, TERT stimulates the proliferation of epidermal progenitor cells, and that function does not require reverse transcriptase activity [53]. Importantly, telomerase affects the expression of neurotrophins, which has a neuroprotective effect and prevents neuronal degradation [54]. Non-canonical functions of telomerase and shelterin proteins deserve close attention as they could provide a link between telomere and cellular processes.

**Figure 2:**
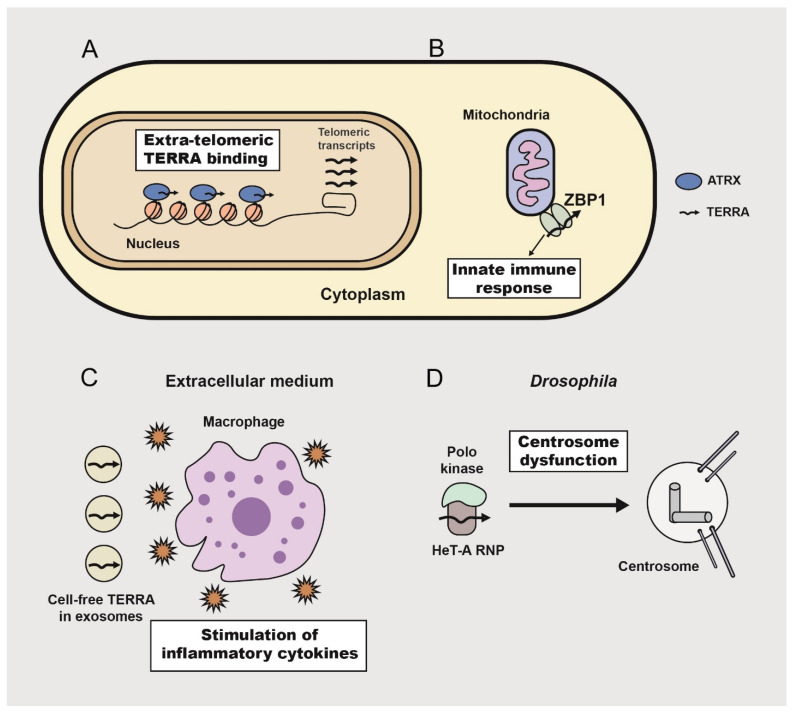
Signaling role of telomeric transcripts induced by overexpression from dysfunctional telomeres. The scheme shows the reported examples of telomeric RNA-mediated regulatory effects. In mammalian cells, TERRA can affect gene expression [60] (**A**) and induce innate immune activation [63] (**B**) or cause inflammatory response [61] (**C**). In the *Drosophila* germline, abundant telomeric ribonucleoprotein particles interact with Polo kinase, affecting centrosome biogenesis during oogenesis and early development [64] (**D**).

Mounting evidence suggests that TERRA is also involved in the telomere signaling pathway (Figure 2). Indeed, TERRA expression is tightly linked to the state of telomeric chromatin [55], changes during the cell cycle [56] and is upregulated in ALT tumor cells [57]. TERRA interacts with a large number of cellular proteins related to different biochemical pathways [34,55,58,59]. An important finding is that TERRA can act in trans as a transcriptional regulator of non-telomeric targets. This regulation is modulated by the antagonistic interactions between TERRA and chromatin remodeler ATRX [60]. TERRA molecules were found in extracellular exosomes during telomere uncapping and telomere shortening [61]. TERRA-containing exosomes were strong inducers of inflammatory response in mammalian cells. The proposed function of this inflammation is to recruit macrophages to eliminate cells with telomeric repeat overexpression, which is the hallmark of telomere disorder [62].

Nassour and co-authors recently reported a novel function of TERRA in the activation of the autophagy program aimed at the elimination of cells with unstable telomeres [63]. This mechanism is mediated by Z-DNA binding protein 1 (ZBP1], a nucleic acid sensor that induces innate immune activation and cell death. Abundant TERRA molecules, which accumulate as a result of telomere dysfunction, bind to ZBP1 leading to the formation of ZBP1 filaments on mitochondria, which triggers autophagy and cell death in human fibroblasts and epithelial cells. These data emphasize the role of TERRA as the messenger of dysfunctional telomeres that triggers cell death. 

An analysis of telomeric RNA biogenesis in the *Drosophila melanogaster* model revealed the relationship between the telomeric transcripts and key protein regulators of the cell cycle [64]. The depletion of the negative regulators of telomeric repeat expression (small RNA pathway component, deadenylase Ccr4 and RNA-binding protein Ars2) led to the overexpression of the main telomeric retrotransposon *HeT-A* in the female germline and the accumulation of *HeT-A* RNPs in the oocyte and early embryos. Aggregates of *HeT-A* RNPs were found near the microtubule-organizing center, MTOC, in the oocyte, and around centrosomes in the syncytial embryos. *HeT-A* overexpression in the germline, caused by the depletion of transcription factors, chromatin components, RNA binding proteins, and small RNA pathway genes, led to phenocopies associated with severe mitotic defects and early embryonic lethality [64,65]. A mass spectrometry analysis of *HeT-A* RNP partners in *Drosophila* syncytial embryos identified key proteins of the cell cycle and centrosome components. The retention of Polo kinase in *HeT-A* RNP aggregates triggered centrosome dysfunction and mitotic catastrophe, leading to early developmental arrest. We found that the overexpression of telomeric repeats in *Drosophila* somatic cells was cytotoxic and also led to the aggregation of *HeT-A* RNPs around centrosomes in mitotic cells. Thus, telomeric RNAs produced by dysfunctional telomeres above the threshold level affect cell cycle machinery and prevent abnormal development. Noteworthily, *HeT-A* RNP-mediated signaling was not associated with telomere shortening or the loss of telomere-capping proteins. In some cases, *HeT-A* overexpression was caused by chromatin changes and a loss of transcriptional silencing in telomeres. However, *HeT-A* telomeric transcripts also accumulated and affected the cell cycle machinery after the depletion of the Ccr4-Not deadenylase complex that mediates the co-transcriptional degradation of telomeric RNAs but does not change the telomeric chromatin state and protection [66]. Given that increasing evidence supports a signaling role for telomeric RNA, the proper coordination of telomeric repeat expression and biogenesis of telomeric RNA becomes more important. 

The depletion of functionally different factors increased the transcription of telomeric repeats in the *Drosophila* germline and showed an embryonic lethality phenotype likely consistent with telomere dysfunction. These data strongly suggest that an activation of transcription in altered but protected telomeres is one of the universal responses of telomeres to various genetic stresses, and telomeric transcripts are able to trigger cell death or developmental arrest to eliminate potentially dangerous cells. This pathway, activated as the response to telomere damage, can be defined as a telomere checkpoint.

It will be interesting to see if TERRA can perform signaling functions in the mitotic spindle of mammalian cells. TERRA extra-nuclear metabolism is not well understood. It was observed that TERRA transcripts form distinct foci in the cytoplasm of tumor cells [67]; however, it is unknown which proteins associate with the TERRA foci. It was observed that shelterin protein TRF1 localized to the mitotic spindle and interacted with Aurora kinase in human cancer cells [68,69]; however, the functional outcome of such a localization remains unknown. It is tempting to speculate that TRF1, which has been shown to interact with TERRA [55], may be bound to TERRA at this location. How dysfunctional but protected telomeres can induce cell cycle arrest in normal mammalian cells remains an open question. Lessons from the fruit fly *HeT-A* RNP signaling mechanism could be helpful in elucidating this pathway.

## 4. Telomeres in Aging: Shortening or Dysfunction?

Alexey Olovnikov proposed in 1973 that telomere shortening is counter to the number of cell divisions [2]. Human fibroblasts’ telomeres do, in fact, shorten with aging, leading to replicative senescence [3], whereas telomerase expression in normal cells results in telomere lengthening and cell immortalization [6,70]. Despite the fact that telomere theory had been supported at the cellular level, Alexey Olovnikov recognized that it was insufficient to explain aging at the organismal level, viewing telomere shortening as a “witness” rather than the underlying cause of aging [71]. 

What causes cell division arrest when telomeres shorten? It is well established that unprotected or critically shortened telomeres induce a persistent DNA damage response leading to p53-mediated cellular senescence or apoptosis [72,73]. This happens because DNA repair mechanisms are inhibited at the telomeres to prevent telomere fusions and inter-chromosome recombination. Shortened telomeres induce persistent DNA damage foci, marked by the phosphorylated histone γH2AX. These foci are termed telomere-associated foci (TAF), and their accumulation is considered to be a consequence of telomere shortening in aging-related pathologies [42,74]. However, cellular senescence in normal organismal aging occurs when telomere length is not critically short. For example, mouse telomere length is around 100 kilobases, and senescence in mouse cells occurs at a telomere length of several tens of kilobases. There is no correlation between telomere length/telomerase activity and the lifespan of rodent species, among which there are extremely long-lived species [75]. Mutations in the genes involved in telomere maintenance (TERC, TERT, POT1, TIN2, TPP) lead to severe syndromes: telomere biology disorders (TBDs) with signs of early senescence [42,76]. However, global telomere shortening is not necessarily observed even in these syndromes. There are TBDs exhibiting excessive telomere elongation or normal telomere length [76]. For example, POT1 mutations associated with longer telomeres caused an increased risk for tumors and blood cancer [77]. Moreover, replicative senescence occurs unequally in cell types with different mitotic capacities since the degree of telomere shortening differs significantly among them. Despite the well-established fact that telomeres do shorten during cell division and aging, these findings suggest that there is no strong causal relationship between telomere length and normal organismal aging, and, indeed, telomere shortening can be considered a “witness” of aging, but not its driver.

Instead, increasing evidence suggests that the accumulation of damage in telomeres, regardless of their length, serves as a signal to trigger cell death. The accumulation of unrepairable damage in telomeres occurs in the tissues of aging primates and mice; noteworthily, the DDR signal is triggered at a normal average telomere length [78,79,80]. Indeed, up to 40% of all DDR foci in the nuclei of aging cells correspond to telomeres, which account for only 0.02% of the genome [79]. Telomere-associated DDR foci have been documented during aging in post-mitotic cells such as neurons, adipocytes, osteocytes, and cardiomyocytes, irrespective of telomere length [81]. One of the striking examples of the negative consequences of length-independent telomere dysfunction is cellular senescence in human melanocytes [82]. Telomere quantitative FISH (Q-FISH) from young and older skin demonstrated that telomeres in melanocytes do not undergo significant shortening with aging, which is also confirmed by previously reported data about the low replicative capacity of differentiated melanocytes [83]. However, during skin aging, an increase in the number of signals of a protein marker of DNA damage, γH2AX, was recorded in the telomeres of melanocytes. Senescent melanocytes were able to induce telomere dysfunction and senescence in neighboring skin cells leading to epidermal atrophy.

Age-related disorders include genomic instability, telomere dysfunction, proteostasis loss, mitochondrial dysfunction, cellular senescence and other metabolic and cellular alterations [39,84]. Many aging-related hallmarks are associated with the generation of DDR in telomeres and, accordingly, the accumulation of TAF. For example, telomere dysfunction has been linked to such signs of aging as mitochondrial dysfunction, chronic inflammation, and loss of proteostasis, suggesting a telomere-centric mechanism of aging [85]. DDR should be able to be activated at both short and long telomeres to promote cellular senescence and aging. It was previously reported that the overexpression of human TERT leading to telomere elongation did not protect fibroblasts from stress-induced senescence [86]. Treatment with general geroprotectors such as rapamycin, 17β-estradiol, and senolytic drugs reduced the percentage of TAF-positive aging cells [87,88,89]. These studies highlight how essential the telomere state is to aging and show that telomeric TAFs can be considered a key hallmark of aging. 

The reasons for length-independent telomere damage may be various influences that change the organization of telomeres, the structure of telomeric chromatin, and the levels of transcription. It was reported that telomeres of normal cells are extremely sensitive to oxidative DNA damage. Oxidative stress in mouse neutrophils led to telomere dysfunction, the accumulation of telomeric DDR signals, and the spread of the senescent phenotype to surrounding cells, without affecting telomere length [90]. Oxidative lesions at the telomeres of normal human fibroblasts and epithelial cells induced replication stress and telomeric DDR activation in the absence of telomere shortening or deprotection [91].

**Figure 3:**
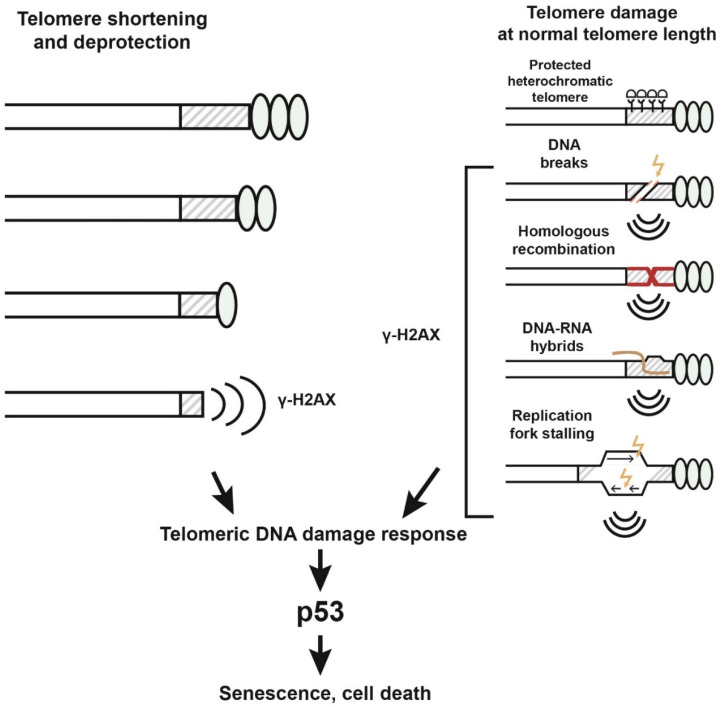
Length-independent internal telomeric DNA lesions lead to DNA damage response. Critical telomere shortening and deprotection both induce cellular DNA damage response (to the left). The scheme to the right shows possible mechanisms for the generation of internal telomeric DNA instability that induces DDR in telomeres irrespective of their length. Broken arrows indicate DNA damage.

Experimental evidence suggests that telomeric repeats are inherently prone to DNA break accumulation (Figure 3). Internal telomeric breaks could be a result of replication-associated defects. In addition, the transcription of telomeric repeats is strongly associated with DNA damage. The formation of TERRA RNA-DNA hybrids (R-loops) in telomeres can lead to the generation of DNA breaks through several mechanisms [33,92,93]. R-loops can cause replication fork stalling and, as a result, DNA breaks and recombination [32,94]. Telomeric R-loops considerably increase homologous recombination events, as discussed in recent extensive reviews [33,95,96,97]. Homologous recombination at the telomeres is inhibited by the chromatin remodeling factor ATRX, TERRA RNA-binding proteins NONO and SFPQ, and other factors that suppress R-loop formation, recombination, and DNA damage in mammalian telomeres [59,97,98].

Internal telomeric DNA breaks, generated in one way or another, serve as substrates for homologous recombination. ssDNA generated as a result of dsDNA break resection can invade the telomeric regions on another chromosome, leading to ectopic recombination and genomic instability. The experimental induction of internal DSBs in mouse telomeres activated homologous recombination and the ALT pathway [99]. Recombination-based telomere elongation is observed in yeast [100,101], ALT cancer cells [57], and in mice in the absence of Pot1 or Rap1 [102,103]. Noteworthily, telomere lengthening during the early embryonic cleavages and in normal somatic tissues in mice is telomerase-independent and requires a recombination-based mechanism [104,105]. Despite the functional role of telomeric DNA breaks in recombination-based telomere maintenance, their levels and appropriate processing appear to be strictly balanced during normal development to prevent the activation of DDR or ALT cell proliferation. The accumulation of telomeric DDR signals in protected, not critically shortened telomeres suggests that this balance is severely impaired in senescent cells and in aging tissues. 

## 5. Nuclear Topology and Epigenetics of Telomeres

The compromised functioning of the nuclear periphery is one of the main factors in the destabilization of telomeres during senescence, laminopathies, and accelerated aging syndromes [106,107,108].

The nuclear envelope plays a pivotal role in the 3D genome architecture. The disruption of nuclear lamina has a global effect on the compaction of chromatin domains and their nuclear positioning [109]. Telomere positioning at the nuclear periphery is a key feature of genome organization in yeast, *Drosophila*, and a subset of mammalian cell types [110,111,112,113]. 

The nuclear periphery provides a safe environment for the stable maintenance of heterochromatin. The inhibition of homologous recombination at the nuclear lamina is a means of suppressing recombination between repetitive sequences [114,115,116]. The main feature of DNA break repair in heterochromatin is that homologous recombination is temporarily blocked and then restarted after relocation to the nuclear periphery where the DSB is isolated from homologous ectopic sequences [117]. Heterochromatin protein 1 (HP1) and histone H3 lysine 9 (H3K9) methyltransferases, typical components of silenced chromatin, are required to prevent Rad51 recruitment to the heterochromatic DNA breaks, and their loss results in the progress of ectopic recombination in *Drosophila* [118]. While the overall mechanism of DNA repair in heterochromatin is unclear, the crucial role of nuclear lamina in this process is a conserved feature. The translocation of heterochromatic DSBs to the nuclear lamina assures their safe reparation in *Drosophila* [118]. In human cells, the activation of the DDR at DSBs and homologous recombination are also inhibited at the nuclear lamina [119]. The integrity of the nuclear periphery compartment and its components ensures the safe repair of repeat-rich regions. The localization of telomeres to the nuclear periphery also represents a way of reducing recombination between highly repetitive telomeric sequences. The localization of yeast telomeres to the nuclear lamina protects telomeric repeats from recombination [120], and the disruption of perinuclear telomere anchoring led to a hyper recombinant telomeric state and a senescent-like phenotype [121]. The global effect of aging on nuclear periphery integrity and heterochromatin structure is clearly linked to a relaxation of DNA repair control. High levels of telomeric DNA damage observed in laminopathies emphasize the central role of telomeres in the global epigenetic changes associated with accelerated aging syndromes [106,107].

Lamins are the main structural proteins associated with the nuclear lamina. Lamins are involved in the DNA repair mechanisms [122] and interact with shelterin proteins [123]. A mutation in the lamin A gene, which produces a shortened form of the protein, causes a premature aging syndrome, Hutchinson–Gilford progeria [124]. Structural defects of lamins also occur during cellular aging, and laminopathy is one of the hallmarks of aging [125,126]. Consistently, factors that associate with lamins and stabilize heterochromatin of repetitive elements delay cellular senescence [127,128,129]. Structural damage of the nuclear lamina leads to systemic effects, the most important of which is a state of globally compromised chromatin epigenetics that includes changes in heterochromatin structure, the misregulation of gene activity, and telomere dysfunction [130,131,132].

Among the various signs of aging, the epigenetic changes are the most dramatic. Aging is associated with the DNA hypomethylation of transposable elements and telomeric regions, as well as the hypermethylation of promoters of coding genes [84,133]. Changes in the DNA methylation pattern in aging have been called an epigenetic clock, owing to their correlation with chronological age. The epigenetic clock of Steve Horvath includes 353 CpG sites in the human genome that methylation changes with age [134,135]. Importantly, the DNA methylation pattern, but not telomere length, is associated with chronological age in humans [136]. It is likely that telomere damage, rather than telomere shortening, is a key sign of aging in a normal organism. In Hutchinson–Gilford syndrome, systemic changes in the epigenetic status of the entire genome are also observed, but telomere dysfunction and TAF accumulation are the main contributors to the manifestation of the disease signs. Telomeropathy is equated with laminopathy in terms of the symptoms of aging [107,137,138]. This point of view is supported by studies of a mouse model of Hutchinson–Gilford syndrome where it was shown that the selective inhibition of the DDR on the telomeres improves tissue homeostasis, reduces inflammation, and prolongs the lifespan of the model animals [139]. 

The effects of lamin B mutation and physiological aging on the telomeres of *D. melanogaster* germ cells are strikingly similar [140]. The appearance of DNA damage foci, enriched in the γH2Av histone variant, and the accumulation of recombination marker Rad51, which are particularly abundant in the telomeres of lamin B mutants and aged flies, can be triggers of the observed germ cell death [140]. RAD51 is a known marker of homologous recombination and break-induced replication [141]. In human cells, RAD51 associates with TERRA and promotes telomeric R-loop formation [92]. The accumulation of recombinase Rad51 at *Drosophila* telomeres in lamin B mutants, as well as in aging germ cells, suggests the activation of homologous recombination between telomeric repeats, which elevates the risk of chromosomal rearrangements [140]. Telomeric chromatin decompaction observed in lamin B mutants can cause defective DNA repair and the accumulation of γH2Av foci. Since the expression of the *Drosophila* telomeric retroelement *HeT-A* is moderately increased in lamin B mutants and in aging, it cannot be excluded that Rad51 associates with *HeT-A* RNAs, thus facilitating homologous recombination and telomere instability [140]. However, the mechanism of the inhibition of homologous recombination in telomeres with the participation of lamin B is still unknown.

Taken together, these data point to a key role for telomere instability in both laminopathy and aging, despite the dramatic changes in genome organization observed during these processes.

## 6. Conclusions

Many lines of evidence suggest that not only telomere shortening, but also length-independent telomere dysfunction, can be considered as “the heel of Achilles of the DNA double helix” [2]. The telomere-centric mechanism of aging implies that telomeres collect information about disorders in the cell and, at a critical level of dysfunction, trigger a response leading to cell death. Such a mechanism, similar in principle to checkpoint systems, explains the key role of telomeres in development and aging. Indeed, only telomeres can provide a universal and rapid response to the enormous range of external and internal stimuli that affect many targets in the cell and pose a threat to genetic stability. The causes of telomere dysfunction can be genetic disorders, external influences, stress of various kinds, physiological aging, laminopathy, and many others. As a result, disruptions in the structure of telomeric chromatin, the appearance of telomeric DNA breaks, replicative stress, and ectopic recombination occur; these serve as signals triggering the telomeric checkpoint, which activates cell cycle arrest and cell death to protect genome stability (Figure 3). This is a fundamental anti-oncogenic mechanism operating in multicellular organisms. However, the upregulation of the DNA damage response system can also strongly stimulate the senescent phenotype, leading to persistent inflammation, a typical characteristic of aging [142]. The molecular nature of length-independent telomere dysfunction and signaling is far from understood. Basically, there are two such mechanisms: internal irreparable damage in telomeres and signals coming from altered telomeres, e.g., telomeric proteins and RNA. The study of telomere signaling is very important because normal aging occurs at an average telomere length; the length of telomeres has been evolutionarily established with an excess and is not physically exhausted in the course of normal development.

Telomere dysfunction and epigenetic changes, primarily those in telomeres, can serve as important biomarkers of processes associated with age-related changes in normal tissues. For example, detecting the level of telomeric DNA methylation associated with the activation of the DDR signal at telomeres can serve to detect signs of premature aging. Telomeric RNAs emanating from non-shortened but functionally compromised telomeres are also an important signature of telomere dysfunction, and TERRA levels can also be considered as a potential diagnostic factor of telomere-associated pathologies. Such multilevel regulation of telomeric homeostasis emphasizes the fundamental importance of this structure in maintaining the integrity of the genome and provides us with potential tools for diagnosing and treating cancer and premature aging.
